# LPA_5_ Is an Inhibitory Receptor That Suppresses CD8 T-Cell Cytotoxic Function via Disruption of Early TCR Signaling

**DOI:** 10.3389/fimmu.2019.01159

**Published:** 2019-05-28

**Authors:** Divij Mathew, Kimberly N. Kremer, Pamela Strauch, Gabor Tigyi, Roberta Pelanda, Raul M. Torres

**Affiliations:** ^1^Department of Immunology and Microbiology, University of Colorado School of Medicine, Aurora, CO, United States; ^2^Department of Physiology, University of Tennessee Health Science Center, Memphis, TN, United States

**Keywords:** CD8 T cells, lysophosphatidic acid, TCR signaling, granule exocytosis, cytotoxicity, inhibitory receptor

## Abstract

Persistent T cell antigen receptor (TCR) signaling by CD8 T cells is a feature of cancer and chronic infections and results in the sustained expression of, and signaling by, inhibitory receptors, which ultimately impair cytotoxic activity via poorly characterized mechanisms. We have previously determined that the LPA_5_ GPCR expressed by CD8 T cells, upon engaging the lysophosphatidic acid (LPA) bioactive serum lipid, functions as an inhibitory receptor able to negatively regulate TCR signaling. Notably, the levels of LPA and autotaxin (ATX), the phospholipase D enzyme that produces LPA, are often increased in chronic inflammatory disorders such as chronic infections, autoimmune diseases, obesity, and cancer. In this report, we demonstrate that LPA engagement selectively by LPA_5_ on human and mouse CD8 T cells leads to the inhibition of several early TCR signaling events including intracellular calcium mobilization and ERK activation. We further show that, as a consequence of LPA_5_ suppression of TCR signaling, the exocytosis of perforin-containing granules is significantly impaired and reflected by repressed *in vitro* and *in vivo* CD8 T cell cytolytic activity. Thus, these data not only document LPA_5_ as a novel inhibitory receptor but also determine the molecular and biochemical mechanisms by which a naturally occurring serum lipid that is elevated under settings of chronic inflammation signals to suppress CD8 T cell killing activity in both human and murine cells. As diverse tumors have repeatedly been shown to aberrantly produce LPA that acts in an autocrine manner to promote tumorigenesis, our findings further implicate LPA in activating a novel inhibitory receptor whose signaling may be therapeutically silenced to promote CD8 T cell immunity.

## Introduction

CD8 T cell function often deteriorates in the presence of prolonged antigen-specific stimulation that accompanies chronic infections and cancer (1). This T cell dysfunction is driven, in part, by signals initiating from inhibitory receptors whose expression are induced upon TCR-mediated activation and that normally function to attenuate antigen-specific CD8 T cell immune responses once pathogen has been neutralized (2). However, in settings of persistent TCR stimulation, CD8 T cell inhibitory receptor expression and signaling is often maintained and exploited by chronic infections and cancer to impair CD8 T cell cytotoxicity and promote T cell exhaustion. Importantly, a number of chronic pathogen infections of global health concern including HBV, HCV, HIV, EBV, and *Plasmodium* promote the development of exhausted CD8 T cells (3–8). Similar to the relative success that checkpoint blockade has enjoyed in the treatment of certain cancers (9, 10), the *in vivo* interference with CD8 T cell inhibitory receptor signaling has led to enhanced immunity during these chronic infections (11, 12).

Cytotoxic T lymphocyte antigen 4 (CTLA-4) and Programmed cell death protein death 1 (PD-1) surface receptors are two of the first identified and characterized inhibitory receptors (13, 14). The *in vivo* therapeutic interference of these inhibitory receptors, referred to as immune checkpoint blockade, is able to restore CD8 T cell function and has achieved success in the treatment of certain cancers (15, 16). Despite these achievements, current checkpoint blockade therapy has been successful for only a minority of patients and a subset of cancers indicating that different cancers use multiple and/or diverse mechanisms to suppress CD8 T cell cytotoxicity and evade anti-tumor immunity. Consequently, there is a strong impetus to identify additional inhibitory receptors to possibly exploit for combination checkpoint blockade therapy in cancer and possibly chronic infections. However, the precise CD8 T cell signaling pathways that are regulated by these inhibitory receptors and the molecular mechanism(s) that restrain CD8 T cell function are not well established for PD-1 and CTLA-4 or other inhibitory receptors currently being considered for immune checkpoint blockade therapy (17, 18). It is evident that a better mechanistic understanding of the signaling pathways and inhibitory mechanisms used by inhibitory receptors will facilitate the targeting of multiple inhibitory signaling pathways and would be expected to lead to enhanced combination checkpoint blockade therapies (18).

Lysophosphatidic acid (LPA) is a bioactive lipid mediator that is generated extracellularly and primarily by the activity of autotaxin (ATX); a secreted phospholipase D enzyme that associates with integrins on the surface of cells where it produces LPA (19, 20). LPA is recognized by 6 different cognate G-protein coupled receptors (GPCRs), known as LPA_1−6_ and acts on various cell types to induce migration, proliferation, cell survival, wound healing, and inflammation (21–25). Notably, levels of both LPA and ATX are often elevated in chronic inflammatory disorders such as chronic viral (HCV and EBV) infections (26–28) autoimmune diseases (29, 30), obesity (31–33), and cancer (21, 23, 27, 34–38). Work from our lab has previously determined that LPA signals via the LPA-5 receptor, LPA_5_, on B cells and CD8 T cells to suppress the antigen receptor-induced calcium response, cell activation, and proliferation *in vitro* and *in vivo* (39, 40). Thus, LPA_5_ functions as an inhibitory receptor on lymphocytes. Together these findings suggest that elevated LPA levels are not only associated with chronic infections and select cancers, but LPA also signals via LPA_5_ to suppress CD8 T cell immunity.

In this report, we characterize specific TCR signaling pathways that are suppressed by LPA_5_ in both mouse and human CD8 T cells and demonstrate that a crucial outcome of this inhibition is impaired cytolytic granule exocytosis and subsequent *in vitro* and *in vivo* cytotoxic function. Thus, these data provide a molecular and biochemical description of how the LPA_5_ inhibitory receptor expressed by CD8 T cells restrains early TCR signaling events to ultimately suppress target cell killing.

## Materials and Methods

### Mice

C57BL/6 (Stock No: 000664, Jackson Laboratories), *Lpar2*^−/−^ mice (41) (gift from Dr. Jerold Chun, Scripps Research Institute), *Lpar5*^−/−^ mice (39), CD45.1 OT-I mice (gift from Ross Kedl, Ph.D., University of Colorado), *Lpar5*^−/−^ OT-I mice, and *Rag2*^−/−^ mice (Stock No: 008449, Jackson Laboratories), *Enpp2*^+/−^ mice (42) (gift from Dr. Susan Smyth, University of Kentucky) were bred and housed at the University of Colorado Anschutz Medical Campus Vivarium (Aurora, CO, USA). OT-I mice (43) harbor CD8 T cells with a transgenic Vβ5Vα2 TCR specific for the SIINFEKL peptide from chicken ovalbumin (residues 257–264) but also recognize SIIGFEKL with lower affinity (44). Splenocytes from *Lpar6*^−/−^ and Nur77^GFP^ OT-I mice (45) mice were gifted from Dr. Iain McKillop (Atrium Health, Carolinas Medical Center University) and Dr. Ross Kedl, respectively. All procedures with animals were approved by the University of Colorado Institutional Animal Care and Use Committee.

### Human Peripheral Blood Mononuclear Cells

Human blood PBMC samples were obtained from two sources. One source was white blood cells isolated via a platepheresis leukoreduction filter chamber from the whole blood of healthy donors donating blood at Bonfils Blood Center (Denver, CO). We received these as de-identified samples. Alternatively, healthy subjects were recruited at the University of Colorado Health and whole blood collected at the Colorado Clinical & Translational Sciences Institute (CTRC) after obtaining written informed consent under a protocol approved by the University of Colorado Institutional Review Board. PBMCs from whole blood were enriched by centrifugations in Ficoll-Paque for 20 min at 2,400 rpm at room temperature. CD8 T cells were enriched using a negative selection CD8 T cell Isolation kit (Miltenyi; 130-096-495). All human studies were performed in compliance with University of Colorado Institutional Review Boards and in accordance with the Declaration of Helsinki.

### Antibodies and Flow Cytometric Analyses

Splenocyte single cell suspensions were counted on a Beckman Coulter Vi-Cell XR prior to staining with fluorochrome conjugated antibodies. Cells were stained in PBS containing 2% BSA and 0.1% sodium azide at room temperature for 30 min. Prepared samples were either analyzed on a Beckman Coulter Cytoflex with CytExpert2.2 software or BD Fortessa using BD FACSDiva software. Digital files were reanalyzed with FlowJo 10.2.1. Below we list the antibodies (and clone names) and source used for this study: CD8 BV421 (53-6.7; Biolegend), CD3 AF647 (145- 2C11; Biolegend), CD44 PerCp Cy5.5 (IM7; Biolegend), I-A/I-E Alexa Fluor700 (M5/114.15.2; Biolegend), CD107 BV421 (1D4B; Biolegend), CD69 PE-Cy7 (H1-2F3, Biolegend).

Human PBMCs were isolated from a ficoll gradient and stained with 10 μg/ml of anti CD3 biotin (Biolegend; OKT3) and subsequently stimulated with avidin for 3 or 5 min followed by fixation with 2% paraformaldehyde. Fixed cells were permeabilized overnight with methanol at −20°C. Cells were washed and stained with anti-p44/42 MAPK (ERK1/2) AlexaFluor 488 (Cell Signaling Tech 13214S) and anti-CD8 AF700 (Biolegend; HIT8a). Mouse splenocytes were stained with 10 μg/ml anti CD3 biotin (Biolegend; 145-2C11) and stimulated with avidin for 3 or 5 min and fixed with 2% paraformaldehyde. After overnight permeabilized in methanol at −20°C, cells were stained with anti-CD8 BV421 (Biolegend, 53-6.7), anti-MHCII AF700 (Biolegend M5/114.15.2), mouse Fc Block (24G2), and anti-p44/42 MAPK (ERK1/2) AlexaFluor 488 (Cell Signaling Tech.13214S) for 45 min at room temperature and analyzed with a BeckmanCoulter CytoFLEX.

### T Cell Peptide Stimulations and Effector Cell Generation

Splenocytes were isolated from OT-I transgenic mice and erythrocytes lysed after incubation with 500 μL 0.83% NH4Cl-Tris Buffer for 5 min at room temperature. OT-I CD8 T cells were stimulated by pulsing splenocytes with 2 μg/mL of SIINFEKL (N4) for 3 days at 37°C. After incubation, cells were washed twice and cultured in fresh media with IL-2 at 20–40 U/mL (Peprotech #200-02) for an additional 3 days. Peptide activated CD8^+^ T cells were isolated after Ficoll separation and negatively enriched with magnetic beads (Miltenyi Isolation Kit; 130-104-075) prior to use. The purity of the negatively enriched CD8 T cell population was typically ~90% or greater. T cell purity was confirmed using antibodies directed against Vα2 APC (Biolegend; B20.1), Vβ5 FITC (Biolegend; MR9-4), and CD8 BV421 (Biolegend; 53-6.7).

### Calcium Mobilization

Splenocytes from C57BL/6 and LPAR-deficient mice were resuspended in 500 μL 0.83% NH_4_Cl-Tris Buffer for 5 min at room temperature to lyse erythrocytes. LPA receptor deficient erythrocyte lysed splenocytes were then stained with eBioscience Cell proliferation Dye eFlour670 (# 65-0840-85) for 10 min at 37°C. Cells were washed several times with media + 5% FBS and mixed 1:1 with B6 splenocytes. Cell mixtures were brought to a concentration of 20 x 10^6^ cells/mL and were loaded with 2.5 μM Indo1-AM (Ebioscience; #65-0856-39) and stained with anti-CD8 PE for 40 min at 37°C. Cells were then washed twice and brought to concentration of 10 × 10^6^ cells/mL in media containing 10 μg/mL anti-CD3 biotin (Biolegend, 145-2C11). Baseline collection was performed for 90 s on a BD LSRFortessa at which point 20 μg/mL avidin was added with or without indicated concentrations of LPA or OTP and LPA_5_ inhibitors. Intracellular calcium levels were collected for another 9 min after stimulation. In some experiments, human CD8 T cells were evaluated for TCR-induced intracellular calcium mobilization after similar Indo-1 loading. In these experiments, human CD8 T cells were treated with 10 μg/ml anti-CD3 (OKT3; Biolegend), baseline measurements collected and then 20 μg/mL avidin was added with or without 10 μM LPA.

### Lipid and OTP Preparation

LPA (18:1) was purchased from Avanti Polar Lipids. Lipids were suspended in organic solution and transferred to glass tubes in 1 mg aliquots. Lipids were lyophilized and sealed in nitrogen and stored at −20°C. For biological use, the lipids were brought to a concentration of 500 μM in media and solubilized for use through sonication. Octadecenyl thiophosphate (OTP) was generated and prepared as previously described (46). Lyophilized OTP was aliquoted to glass tubes and tubes were purged of air with nitrogen. For biological use, OTP was suspended in media and further solubilized through sonication. LPA and OTP were both used for *in vitro* experiments. However, as a metabolically stable LPA analog (47, 48), OTP also associates relatively selectively with LPA_5_ (49) and was typically used in longer *in vitro* experiments. The UA-02-05 LPA_5_ inhibitor was a kind gift from Dr. Marc Nazare (Leibniz-Institut für Molekulare Pharmakologie FMP, Germany) and was stored in DMSO at 10 mM concentrations at −20°C. Working concentrations of 10 μM were used for inhibition of human and mouse LPA_5_ receptor signaling.

### *Ex vivo* Stimulation

Splenocytes were lysed of erythrocytes and stained with 10 μg/mL of anti-CD3 biotin (Biolegend, 145-2C11) for 10 min at 37°C. Cells were then stimulated with 20 μg/mL avidin for 3 or 5 min and terminated with 2% paraformaldehyde. Fixed cells were then permeabilized overnight with methanol at −20°C. The next day, cells were washed and stained with antibodies (and conjugated fluorochromes) to CD8 BV421, CD4 APC, MHCII AF700, and p44/42 MAPK (ERK1/2) Alexa Flour 488 (Cell Signaling Tech., 13214S) for 45 min at room temperature and analyzed with a Beckman Coulter CytoFLEX.

### Real Time Quantitative PCR

Isolated CD8 T cells from pooled spleens and lymph nodes were used for the analysis of LPA receptor gene expression in naïve lymphocytes. Day 6 peptide activated OT-I CD8 T cells were used to determine changes in LPA gene expression in effector T cells. Relative qPCR was performed on a Roche LightCycler 480 with Power SYBR Green PCR Master Mix (ThermoFisher #4367659). Reactions were held at 95°C for 10 min, and then cycled between 95°C (15 s) and 60°C (60 s) 40 times. Data were normalized to 18S rRNA where the sequence is cross-reactive to mouse and human. Relative expression of *Lpar1-6* and *LPAR1-6* were normalized to 18S rRNA. Sequences for primers used to detect *Lpars, LPARs* and *18S* mRNA were synthesized by Integrated DNA Technologies (Iowa) as listed below.

Mouse primer sequences:

*Lpar1* (FW: *CTATGTTCGCCAGAGGACTATG*, Rev*: GCAATAACAAGACCAATCCCG)*

*Lpar2* (FW: *CACACTCAGCCTAGTCAAGAC*, Rev: *GTACTTCTCCACAGCCAGAAC*)

*Lpar3* (FW: *ACCAACGTCTTATCTCCACAC*, Rev: *CAGTTCAGGCCGTCCAG*)

*Lpar4* (FW: *AGGATGGAGTCGCTGTTTAAG*, Rev: *CTAACTTCCTCTTGGATAGCTGG*)

*Lpar5* (FW: *TGGAGGTGAAAGTCATGCTC*, Rev: *GTATCTCGATAGRCAGGGCAC*)

*Lpar6* (FW: *CACATCTGAATAGCAAAGGCG*, Rev: *TGAACATGCACCCGTACAG*)

18S rRNA (FW: *GGGAGCCTGAGAAACGGC*, Rev: *GGGTCGGGAGTGGGTAATTT*)

Human primer sequences:

*LPAR1* (FW: *AATCGGGATACCATGATGAGTCTT*, REV: *CCAGGAGTCCAGCAGATGATAAA*)

*LPAR2* (FW: *CAGCCTGGTCAAGACTGTTGT*, REV: *TGCAGGACTCACAGCCTAAA*)

*LPAR3* (FW: *ACGGTGATGACTGTCTTAGGG*, REV: *CACCTTTTCACATGCTGCAC*)

*LPAR4* (FW: *AAAGATCATGTACCCAATCACCTT*, REV: *CTTAAACAGGGACTCCATTCTGAT*)

*LPAR5* (FW: *CGCCATCTTCCAGATGAAC*, REV: *TAGCGGTCCACGTTGATG*)

*LPAR6* (FW:*GGTAAGCGTTAACAGCTCCCACT*, REV: *TTTGAGGACGCAGATGAAAATGT*).

### *In vitro* CD8 T Cell Cytotoxicity Assay

B16.cOVA or B16.F10 (50) cells were transduced with the IncuCyte NucLight Red Lentivirus Reagent (Essen Bioscience; #4474) and selected with 2 μg/mL puromycin. Stable B16.cOVA.RFP cells were maintained in culture with 750 μg/mL G418 (for selection of OVA expression) and 2 μg/mL puromycin (for selection of RFP expression), while stable B16.F10.RFP cells were cultured with only 2 μg/mL puromycin. B16 tumor cells (1 × 10^4^) were plated in each well of a 96 well flat bottom plate at 37°C and after overnight culture and tumor cell adherence, 10^5^ peptide stimulated OT-I CD8 T cells were added together with the IncuCyte Caspase-3/7 Green Apoptosis Assay Reagent (Essen Bioscience; #4440). To demonstrate antigen specificity, B16.cOVA tumor cells were also plated with naïve or TCR-activated P14 CD8 T cells where the P14 transgenic TCR is specific for LCMV gp33–41 (gift from Dr. Ross Kedl). Specific tumor cell killing was then identified as RFP+Green+ cells and monitored over time capturing images every 2 h over a 24 h period using the IncuCyte live-cell analysis system. Tumor cell death is represented as normalized tumor cell death calculated as total RFP+Green+ cells in the presence of OT-I CD8 T cells/total RFP+ cells. In some experiments, we tested how antigen affinity for the TCR influenced tumor cell killing using a relatively low (SIIGFEKL; K_D_~10 μM) or high (SIINFEKL; K_D_~5 μM) affinity peptide specific for the OT-I TCR (44). Thus, B16.F10.RFP cells were peptide pulsed overnight, washed and effector OT-I CD8 T cells added and immediately imaged every 2 h for 24 h. To test human CD8 T cell cytolytic function, human CD8 T cells were plated 10:1 with the human triple negative breast cancer line SUM.159.RFP (51) (gift from Dr. Jennifer K. Richer, University of Colorado). Alloreactive cytotoxicity against SUM.159.RFP line was measured every 2 h for 96 h.

### Granule Exocytosis Assays

For detection of granule export to the cell surface, B16.cOVA tumor cells were co-cultured with peptide activated T cells for 4 h after staining with anti-CD107a BV421 (Biolegend, clone ID4B) in media alone or in the presence of OTP. Granzyme and perforin levels were evaluated by pretreating OT-I CD8 T cells with Brefeldin A (5 μg/ml) prior to co-cultures. After 4 h, cells were fixed with 2% paraformaldehyde and permeabilized with Invitrogen Permeabilization Buffer (#1960027) and stained with anti-perforin APC (clone eBioOMAK-D) and anti-Granzyme B FITC (clone GB11). All samples were analyzed on a Beckman Coulter CytoFLEX.

To measure perforin localization, B cells were negatively-enriched using magnetic anti-CD43 beads (Miltenyi Biotec) and loaded with 2 μg/ml of SIINFEKL peptide for 2 h at 37°C and washed twice. Subsequently, 3 × 10^5^ peptide-loaded B cells and 3 × 10^5^ previously activated OT-I T cells were mixed and centrifuged at 500 rpm for 5 min in serum-free medium, then 20 μM OTP was added to the indicated samples followed by incubation at 37°C for 20 min prior to plating on Poly-L-Lysine-coated (Sigma) coverslips. Cells were then fixed with 4% paraformaldehyde in PBS, permeabilized with 0.15% Triton Surfact-Amps (Thermofisher), Fc receptor blocked, then stained with APC-conjugated anti-perforin, PE-conjugated anti-B220, Flash Phalloidin Green 488 (Biolegend), and cured with Prolong Diamond anti-fade with DAPI (Invitrogen). Cells were imaged using an Eclipse TE2000-E (Nikon) with a 100x oil-immersion objective, captured with SlideBook6 software (3i, Intelligent Imaging Innovations, Inc.), and analyzed with ImageJ/FIJI (ImageJ open source software, http://Imagej.net). To assess perforin localization, FIJI was utilized to assess the pixel intensity of perforin across a Region of Interest that originated at the point of actin polymerization (0 μm) and encompasses the entire cell. An OT-I T cell was scored positive for perforin localization to the synapse if a majority of perforin intensity occurred in the proximal half of the cell from the synapse.

### *In vivo* Cytotoxicity Assay

C57BL6 mice or *Enpp2*^+/−^ were immunized intraperitoneally with 40 μg of anti-CD40 (FGK4.5), 40 μg of polyinosic-polycytidylic acid (pI:C), and 150 μg OVA for expansion of OVA reactive CD8+ T cells (52). Four days after immunization, immunized host mice were intravenously injected with target cells. Target cells were a 1:1 mix of 0.5 μM eFlour760 stained splenocytes and 5 μM eFlour670 stained splenocytes. The 0.5 μM eFlour760 stained cells were pulsed with 2 μg/mL SSIEFARL (irrelevant peptide derived from herpes simplex virus) and the 5 μM eFlour670 stained cells were pulsed with 2 μg/mL of SIIGFEKL or SIINFEKL peptides for 1 h at 37°C prior to immunization. One day after immunization of target cells, the spleens of host mice were recovered and analyzed for the loss of target cells.

### *In vivo* Tumor Assays

C57BL6 mice were restrained in the supine position and injected with 1 × 10^5^ B16.cOVA tumor cells into the hind right leg subcutaneously. Five days post tumor transfer when tumors were palpable, 5 × 10^5^ wild type OT-I or *Lpar5*^−/−^ OT-I T cells were transferred intravenously into tumor-bearing hosts and tumor weight measured 15 days post T cell adoptive transfer.

The EG7 lymphoma cell line (53) was obtained from ATCC (CRL-2113). EG7 tumors were established by the subcutaneous injection of 1 × 10^5^ EG7 cells into the flank of either *Rag2, Lpar5*^−/−^, or WT mice. Tumor weight was measured from excised tumors after sacrifice and 12 days post tumor challenge.

### Statistical Analyses

Statistical analysis was performed on GraphPad Prism software version 5.2 using either a one- or two-tailed unpaired Student's *t*-test or two-Way ANOVA.

## Results

### LPA_5_ Is Expressed by Human CD8^+^ T Cells and Suppresses TCR-Induced Intracellular Calcium Mobilization and ERK Activity

**Figure 1:**
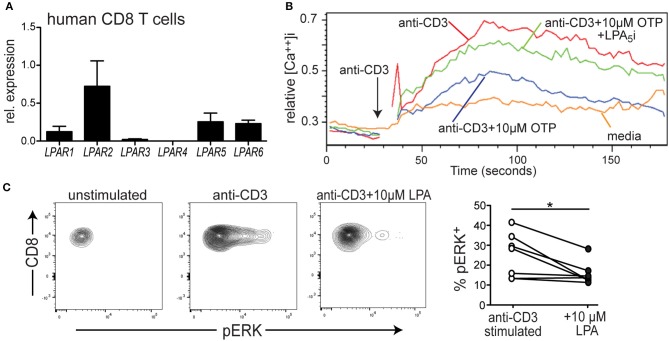
LPA_5_ is expressed by human CD8 T cells and signals to inhibit TCR-induced ERK activity and increased cytosolic calcium levels. **(A)** Expression of *LPAR1-6* by purified human CD8 T cells isolated from the peripheral blood of 3 healthy donors. qPCR expression analyses were normalized to 18 s rRNA. **(B)** Intracellular calcium levels over time in human CD8 T cells stimulated with media alone (orange), anti-CD3 alone (red), or with 10 μM OTP (blue) or in the presence of 10 μM OTP and the small molecule UA-02-85 LPA_5_ inhibitor (LPA_5_i; green). PBMCs isolated from healthy donors were loaded with indo1, stained with anti-CD8 and stimulated with 10 μg/mL biotin-conjugated anti-CD3 + avidin. Data is representative of 3 independent experiments from 2 healthy donors. **(C)** Representative examples of pERK levels examined by flow cytometric analyses in untreated CD8 T cells or after 3 min of treatment with 10 μg/mL of biotin conjugated anti-CD3 and avidin in the absence or presence of 10 μM LPA. Panel on right summarizes data accumulated from 3 different donors. *p < 0.05 by Student's *t-*test. Methanol permeabilized cells were stained for CD8 and pERK and gated on CD8+ T cells.

We have previously reported that naïve murine CD8 T cells express the LPA_2_, LPA_5_, and LPA_6_ GPCRs and that LPA_5_ was able to negatively regulate mouse CD8 T cell TCR signaling (39). Whether human CD8 T cell TCR signaling is similarly subject to LPA_5_ regulation is not established so we initially determined if human CD8 T cells expressed the same orthologous LPA receptors using qPCR analysis of CD8 T cells isolated from peripheral blood. We note that an antibody recognizing surface expression of human (or mouse) LPA_5_ is not currently commercially available. The results from these qPCR analyses show that, similar to naïve mouse CD8 T cells, human CD8 T cells predominantly express *LPAR2, LPAR5*, and *LPAR6* but also harbor lower amounts of *LPAR1* and *LPAR3* (Figure 1A).

**Figure 2:**
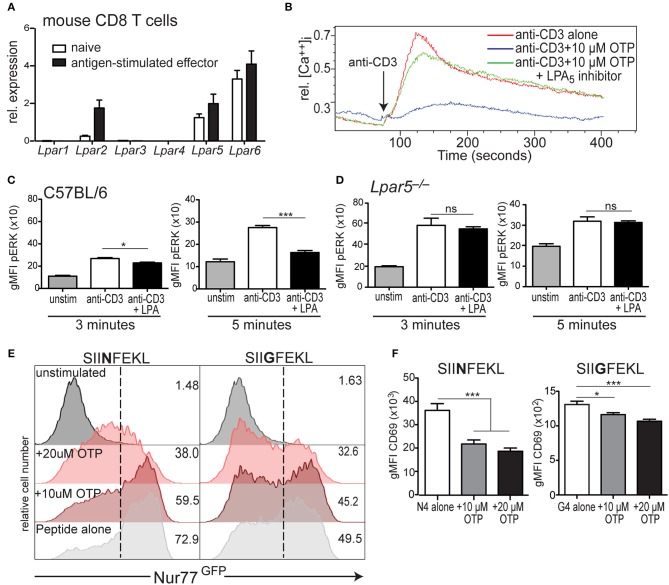
LPA_5_ is responsible for negatively regulating murine CD8 T cell early TCR signaling. **(A)** Expression of *Lpar1-6* by naïve and antigen-stimulated OT-I CD8 T cells. Data is accumulative for 3–4 analyses of CD8 T cells purified from naïve or peptide-activated OT-I splenocytes. **(B)** Intracellular calcium levels over time in murine CD8 T cells stimulated with anti-CD3 alone (red) or anti-CD3 in the presence of 10 μM OTP (blue) or in the presence of 10 μM OTP and the small molecule UA-02-85 LPA_5_ inhibitor (LPA_5_i; green). **(C)** C57BL/6 and **(D)** *Lpar5*^**−/−**^ CD8 T cells were left untreated (gray) or treated with biotin-coupled anti-CD3 and subsequently stimulated with avidin for either 3 min (left) or 5 min (right) alone (white) or in the presence of 10 μM LPA (black) and intracellular pERK levels measured by flow cytometric analyses. Data are representative of technical triplicates and similarly observed in 4 independent experiments. *p < 0.05; ***p < 0.0005; ns, not significant as determined by one-tailed paired Student's *t-*test. **(E)** Nur77^GFP^ expression by WT OT-I Nur77^GFP^ CD8 T cells stimulated with 2 μM of SIINFEKL (N4; left panels) or SIIGFEKL (G4; right panels) peptide for 24 h alone (light gray) or in the presence of 10 μM OTP (dark red) or 20 μM OTP (light red). Data are representative of two independent experiments. **(F)** CD69 expression by OT-I CD8 T cells stimulated with 2 μM of SIINFEKL (N4; left panels) or SIIGFEKL (G4; right panels) peptide alone (white) or in the presence of 10 μM (gray), 20 μM (black) OTP. *p < 0.05; ***p < 0.005 as determined by one-tailed paired Student's *t-*test. Data is accumulative of two independent experiments done with technical triplicates.

To directly test whether LPA also suppressed TCR signaling by human CD8 T cells, PBMCs were collected from healthy human donors, loaded with the indo-1 ratiometric calcium-sensitive dye and T cells were stimulated with biotin-labled anti-CD3 and subsequently cross-linked with avidin. Figure 1B reveals that treatment of human CD8 T cells with anti-CD3 + avidin leads to a substantial increase in cytosolic calcium that is significantly suppressed in the presence of a metabolically stable LPA analog, OTP (47, 48). Notably, OTP is a potent LPA_5_ agonist that associates with LPA_5_ at an EC_50_ that is 100-fold reduced compared to any other LPARs (49) suggesting OTP can be considered a relatively selective LPA_5_ agonist. Thus, these data show that TCR-induced increase of intracellular calcium by human CD8 T cell is significantly inhibited in the presence of 10 μM OTP (Figure 1B), similar to that observed with murine T cells (Figure 2B and Supplemental Figure 1A). To provide further support that this inhibition of TCR signaling was mediated by LPA_5_, we tested the ability of human CD8 T cells to increase intracellular calcium levels after TCR stimulation with OTP and in the presence of a small molecule inhibitor specific for human (and mouse) LPA_5_ [UA-02-85; (54)]. These results demonstrate that in the presence of the UA-02-85 LPA_5_-specific antagonist, the suppression of TCR-induced increase in intracellular Ca^2+^ was severely attenuated (Figure 1B). Together, these data strongly suggest that upon recognition of its lipid ligand, LPA_5_ also signals to negatively regulate TCR-mediated calcium release from intracellular stores in human CD8 T cells.

To address whether LPA_5_ regulated additional TCR-induced signaling pathways, we examined ERK activity as measured by the presence of phosphoERK (pERK) after anti-CD3 mediated TCR signaling alone or in the presence of 10 μM LPA. Here, human CD8 T cells isolated from PBMCs were stimulated with biotinylated anti-CD3 and cross-linked with avidin for 3 min and in the absence or presence of 10 μM LPA and pERK levels and percent pERK+ cells measured by flow cytometric analyses. These results (Figure 1C) show that in the presence of LPA not only were the levels of pERK diminished but also the frequency of human CD8 T cells harboring pERK was significantly reduced after TCR stimulation. Together, these results reveal that LPA_5_ signaling by human CD8 T cells suppresses TCR-induced increases in intracellular calcium levels and ERK activity.

### LPA_5_ Is Expressed by naïve and Effector Murine CD8 T Cells and Disrupts Early TCR Signaling

Naïve CD8 T cells express the LPA_5_ inhibitory receptor, in contrast to the PD-1 and CTLA-4 inhibitory receptors whose expression is absent on naïve T cells but is induced upon TCR-mediated activation (18). Thus, we asked if LPA_5_ was also differentially expressed by effector CD8 T cells. To accomplish this, we used OT-I CD8 T cells that express the OT-I TCR transgene specific for the SIINFEKL chicken ovalbumin (OVA) peptide (43). Naive OT-I CD8 T cells were stimulated with cognate peptide, SIINFEKL, for 3 days and rested an additional 3 days in the presence of IL-2 followed by qPCR analysis of *Lpar* expression. These results (Figure 2A) demonstrate that peptide-activated OT-I CD8 T cells express the same LPA receptors as naïve cells and at similar levels for LPA_5_ and LPA_6_ whereas LPA_2_ expression is considerably higher on activated effector CD8 T cells relative to naïve cells (Figure 2A).

We have previously shown that the inhibition of TCR-induced mobilization of intracellular calcium by CD8 T cells was dependent on LPA_5_ and not LPA_2_ (39). However, in that study we did not rule out a possible role for LPA_6_, which is abundantly expressed by naïve and effector CD8 T cells (Figure 2A). Thus, to unequivocally establish LPA_5_ as the LPA receptor able to restrain TCR signaling, we assayed the ability of LPA to inhibit TCR-induced calcium signaling through analyses of CD8 T cells singly deficient for LPA_5_, LPA_2_, or LPA_6_. These analyses document that LPA inhibition of TCR signaling is regulated uniquely by LPA_5_ and that neither LPA_2_ nor LPA_6_ appear to contribute to LPA suppression of TCR mediated signaling (Supplemental Figure 1A). This conclusion was further supported by the ability of the LPA_5_-specific small molecule inhibitor [UA-02-85; (54)] to prevent LPA_5_-mediated suppression of TCR-induced intracellular calcium mobilization by mouse CD8 T cells (Figure 2B). Thus, genetic and pharmacological approaches clearly demonstrate that LPA_5_ inhibitory signaling is responsible for attenuating TCR-induced CD8 T cell signaling by both human and mouse CD8 T cells.

### LPA_5_ Signaling by CD8 T Cells Disrupts TCR-Induced ERK Activation, Nur77 and CD69 Expression

As observed with human CD8 T cells, when isolated C57BL/6 CD8 T cells were stimulated via the TCR in the presence of 10 μM LPA, pERK levels were significantly reduced ~15% 3 min later and even further reduced ~40% at 5 min after stimulation compared to vehicle-treated cells (Figure 2C). LPA-mediated inhibition of ERK activation was also dependent on the LPA_5_ inhibitory receptor as TCR-stimulation of *Lpar5*^−/−^ CD8 T cells resulted in equivalent levels of pERK independent of the presence of LPA (Figure 2D). In addition, these results (Figure 2D) further reveal that the basal (unstimulated) pERK levels in *Lpar5*^−/−^ CD8 T cells are significantly increased compared to wild type CD8 T cells (^*^p < 0.05) and that 3 min after anti-CD3 stimulation pERK levels are further significantly increased (^***^p < 0.0005) compared to anti-CD3 treated wild type CD8 T cells whereas after 5 min elevated pERK levels in *Lpar5*^−/−^ CD8 T cells are comparable to wild type levels.

*Nr4a1* is an immediate early response gene that encodes Nur77 and whose expression is quickly up-regulated after antigen receptor signaling (45, 55). Nur77^GFP^ OT-I mice harbor a gene encoding GFP in the *Nr4a1* locus allowing GFP to be used as a surrogate for Nur77 expression. When Nur77^GFP^ OT-I CD8 T cells were stimulated for 24 h by peptide-pulsed splenocytes in the presence of OTP, Nur77 expression was inhibited in a dose dependent manner to both SIINFEKL (N4) peptide or a lower affinity variant, SIIGFEKL (G4) peptides (Figure 2E). Importantly, the viability of CD8 T cells after 24 h treatment with 10 μM OTP was unaffected and reduced to approximately 80% with 20 μM OTP treatment (Supplemental Figure 1B). Finally, we evaluated how LPAR signaling affected the expression of the CD69 surface activation antigen by OT-I CD8 T cells 24 h after stimulation with SIINFEKL or SIIGFEKL-pulsed splenocytes. These results revealed that in the presence of OTP, CD69 expression was significantly inhibited after TCR-mediated activation using either a relatively high or low affinity peptide ligand (Figure 2F) and with a similar dose dependency (Supplemental Figure 1C). Together our findings show that LPA-LPA_5_ signaling impedes TCR-induced intracellular calcium mobilization and ERK activation in human and mouse CD8 T cells and suppresses Nur77 expression and expression of the CD69 T cell activation antigen by murine CD8 T cells.

### LPA Signaling Impairs *in vitro* CD8 T Cell Cytotoxicity

**Figure 3:**
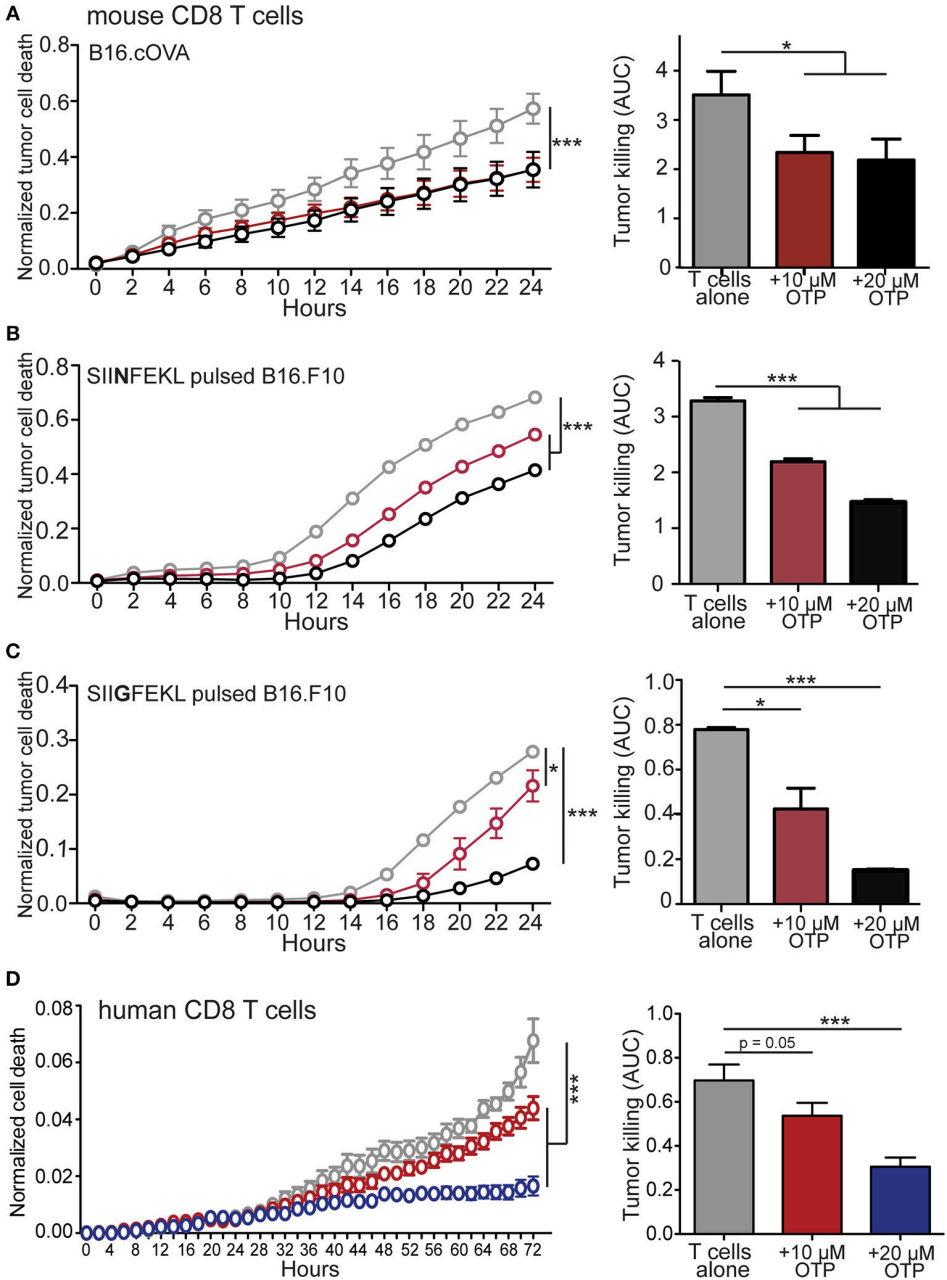
LPA inhibits *in vitro* murine and human CD8 T cell cytotoxic function. **(A)** Antigen activated effector OT-I CD8 T cells were added to RFP+ B16.cOVA cells together with a cell-permeable green fluorescent caspase-3/7 substrate and apoptotic (green+) RFP+ tumor cell cytotoxicity measured over time in the absence (gray) or presence of 10 μM (red) or 20 μM (black) OTP. Bar graphs quantify total B16.cOVA cell death over the 24 h period as area under the curve (AUC). ***p < 0.005 using two-way ANOVA or *p < 0.05 using Student's *t-*test. In **B,C** parental B16.F10.RFP tumor cells (not expressing cOVA) were plated and treated with 2 μg/mL of either **(B)** SIINFEKL high affinity peptide or **(C)** SIIGFEKL low affinity peptide for 24 h prior to the addition of activated OT-I T cells and tumor cell killing measured over 24 h. *p < 0.05; ***p < 0.0005 using a one-tailed paired Student's *t-*test *or* two-way ANOVA. Data are representative of 3 independent experiments with 3 technical replicates per group. **(D)** CD8 T cells enriched from PBMCs isolated from a health donor were added to SUM159.RFP tumor cells and RFP+ tumor cell killing monitored over time in the absence (gray) or presence of 10 μM OTP (red), or 20 μM OTP (blue). ***p < 0.005 using two-way ANOVA. Area under the curve is calculated for media only, media + 10 μM OTP, and media + 20 μM OTP. Data is accumulation of 4 different donors with 4 captured images per well and 3 technical triplicates per condition. ***p < 0.0005 by Student's *t*-test.

The ability of CD8 T cells to exert cytotoxic activity is critically dependent on both intracellular calcium mobilization and ERK activation (56–61) and we find that LPA_5_ signaling impairs both of these events upon TCR stimulation of both human and mouse CD8 T cells (Figures 1, 2). In a mouse melanoma model we have reported that melanoma-specific *Lpar5*^−/−^ CD8 T cells are better able to control tumor growth compared to wild type melanoma-specific CD8 T cells (39). We had attributed the heightened tumor control by LPA_5_-deficient CD8 T cells to increased numbers of tumor-specific CD8 T cells that are generated after TCR-mediated activation in the absence of this inhibitory receptor. However, the ability of LPA to suppress CD8 T cell cytotoxic function was not directly evaluated. Given the established role of both intracellular calcium and ERK activity to cytotoxic function, we next tested if LPA signaling also regulated CD8 T cell effector function using an *in vitro* live cell imaging system to measure antigen-specific CD8 T cell cytotoxicity. Specifically, B16.cOVA and parental B16.F10 cells were stably transduced with an RFP reporter to allow quantification of RFP+ target cells (Supplemental Figure 2A). To measure CD8 T cell tumor cytotoxicity, we measured tumor apoptotic cell death with the use of a fluorescently-labeled substrate for activated caspase 3/7 (Supplemental Figure 2B). This *in vitro* system was characterized to define optimal cell concentrations, effector to target ratios and to confirm the requirement for antigen-specific TCR stimulation of CD8 T cells prior to cell killing (Supplemental Figures 2C–E). To directly test if LPA was able to modulate CD8 T cell cytotoxicity, we generated OT-I effector cytotoxic lymphocytes (CTLs) *in vitro* by stimulating OT-I CD8 T cells with SIINFEKL loaded antigen presenting cells as described and prior to culturing with the B16 melanoma target cells. In initial experiments antigen-activated OT-I CD8 T cells were transferred to cultured RFP+ B16.cOVA cells along with the caspase 3/7 detection reagent, and tumor cell death was monitored over time as RFP+Casp3/7+ in the presence or absence of 10 or 20 μM OTP. In these experiments, B16.cOVA tumor cells constitutively express, process, and present the ovalbumin-derived high affinity SIINFEKL peptide in the context of the MHC-I receptor to CD8 T cells. Thus, the SIINFEKL peptide serves as a surrogate tumor antigen and “tumor-specific” OT-I CD8 T cells initiate tumor killing within 2 h (Figure 3A). Importantly, at 24 h this antigen-specific CD8 T cell cytotoxicity was significantly suppressed by both concentrations of OTP to <66% of the level of killing in the absence of OTP (Figure 3A). We next evaluated whether LPA inhibition of CD8 T cell killing ability was influenced by antigen affinity for TCR. To accomplish this, RFP+B16.F10 parental cells were plated and pulsed for 24 h with either the relatively high affinity SIINFEKL (N4) native peptide or the lower affinity SIIGFEKL (G4) peptide (62, 63), washed and effector OT-I CD8 T cells added prior to imaging. The results from these experiments showed that tumor killing by OT-I CD8 T cells was slightly delayed in response to the weaker affinity SIIGFEKL relative to SIINFEKL stimulation of OT-I CD8 T cells (Figures 3B,C). Importantly, addition of 10 or 20 μM OTP significantly reduced antigen-specific tumor killing and appeared to suppress low affinity SIIGFEKL stimulation (>80% reduction) more than high affinity SIINFEKL stimulation (55% reduction). OTP treatment alone of B16.F10.RFP cells did not induce cell death (Supplemental Figure 2F) confirming melanoma cell death was induced by CD8 T cell cytotoxicity. Thus, these data strongly indicate that LPA impairs tumor killing by CD8 T cells following recognition of cognate high affinity antigens and physiologically relevant low affinity antigens.

To assess if functional interference of LPA_5_ signaling by human CD8 T cells also could inhibit target cell killing, we used our *in vitro* killing assay with SUM159.RFP human breast cancer cells stably transfected with RFP as target cells. Consistently, we find that the addition of 10 and 20 μM OTP suppressed allogeneic mediated cytotoxicity by human CD8 T cells in a dose-dependent manner (Figure 3D). Thus, the LPA_5_ expressed by both human and mouse CD8 T cells engages LPA to inhibit both human and murine T cell TCR signaling, activation and cytotoxicity.

### LPA_5_ Signaling Impairs *in vivo* CD8^+^ T Cell Cytotoxicity and Loss of LPA_5_ Improves Tumor Control

**Figure 4:**
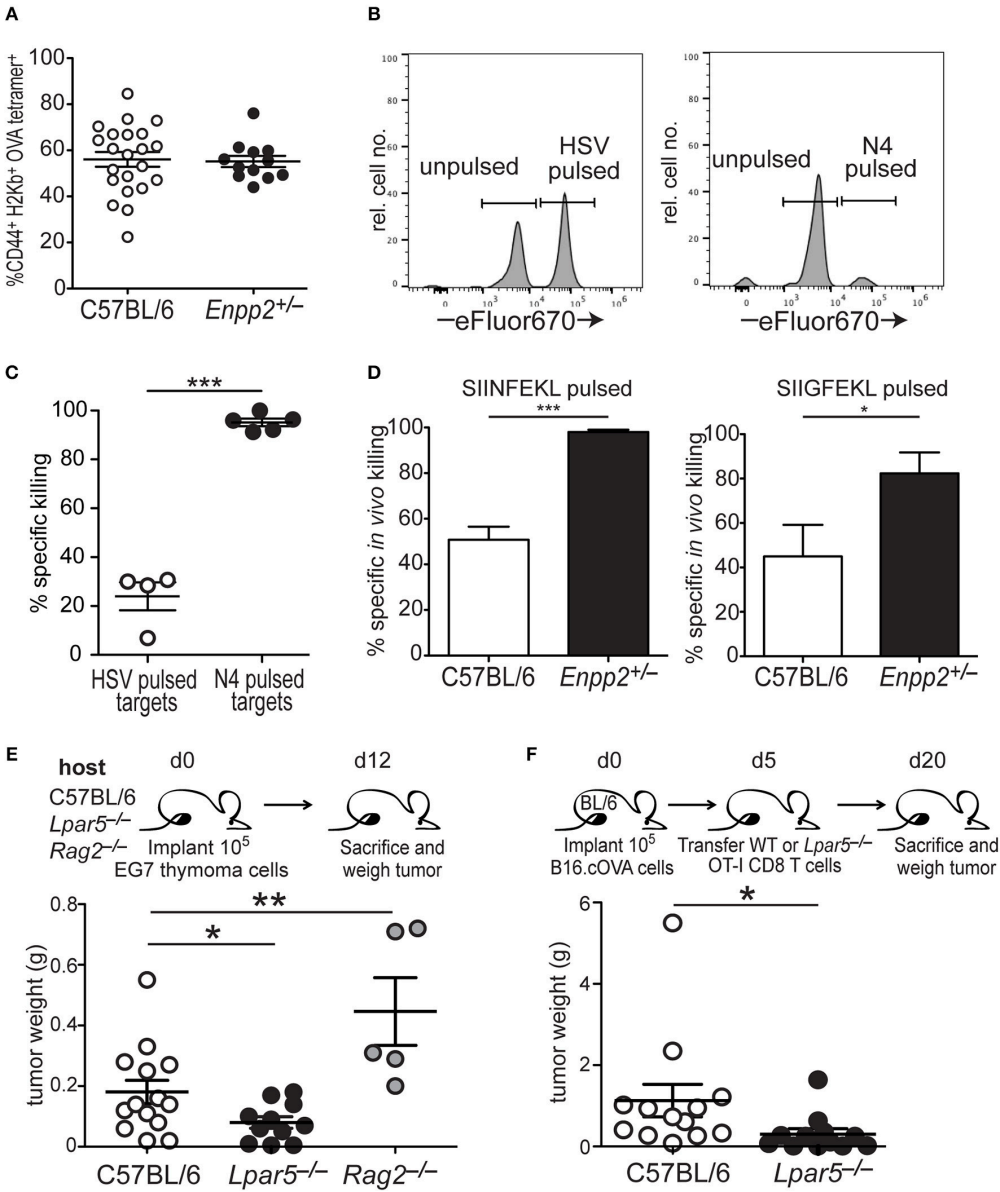
LPA signaling via LPA_5_ suppresses *in vivo* antigen-specific CD8 T cell cytotoxicity. **(A)** Frequency of OVA-reactive endogenous CD8 T cells (CD44+H2-K^b^-OVA tetramer+) in the spleens of C57BL/6 (open) or *Enpp2*^+/−^ (filled) mice 4 days after immunization with pI:C, anti-CD40 and OVA. **(B)** Panels show histograms of representative target cell killing 1 day after the transfer of target cells pulsed either with an irrelevant (HSV; left panel) or antigen-specific (OVA; right panel) peptide. Peptide-pulsed splenocytes were stained with a relatively high concentration of eFluor670 whereas unpulsed splenocytes are stained with a relatively low eFluor670 concentration. **(C)** Percent specific killing of irrelevant (HSV) peptide-pulsed splenocytes (open circles) and SIINFEKL (N4) peptide-pulsed splenocytes (filled circles) by endogenous SIINFEKL-specific CD8 T cells 4 days after immunization with anti-CD40, pI:C, and OVA. Percent specific killing was calculated as: (1– experimental ratio/pre-transfer ratio) × 100 and where the Ratio = %peptide–pulsed cells/% non-pulsed cells. ***p < 0.0005 using a one-tailed paired Student's *t-*test. **(D)** Percent antigen-specific killing of SIINFEKL-pulsed (left) or SIIGFEKL-pulsed (right) target cells 24 h after i.v. transfer of target cells into immunized mice. Data are cumulative of two experiments with 4–10 mice per group. *p < 0.05; ***p < 0.0005 using a one-tailed paired Student's *t-*test. **(E)** EG7 lymphoma cells were implanted into either WT, *Lpar5*^**−/−**^, or *Rag2*^**−/−**^ hosts for 12 days after which they were isolated and weighed. Data are cumulative of 3 independent experiments reflecting tumors recovered from 13 WT hosts, 11 *Lpar5*^**−/−**^ hosts, and 5 *Rag2*^**−/−**^ hosts. *p < 0.05; **p < 0.005 using a one-tailed paired Student's *t-*test. **(F)** B16.cOVA tumor cells were implanted into the flank of B6 mice for 5 days after which either wild type or *Lpar5*^**−/−**^ OT-I CD8 T cells were transferred i.v. into tumor bearing hosts. Fifteen days later recipients were sacrificed and tumors resected and weighed. *p < 0.05; data represents two independent experiments with 12–13 mice per group.

CD8 T cell cytotoxic activity is significantly impaired *in vitro* in the presence of OTP (Figure 3). To provide *in vivo* confirmation of these findings, we exploited mice heterozygous for the *Enpp2* gene (*Enpp2*^+/−^), which encodes ATX, the extracellular enzyme responsible for generating the vast majority of extracellular LPA (64–66). Although *Enpp2*^−/−^ homozygous mice are embryonic lethal due to vascular and nervous system abnormalities (67, 68), heterozygous *Enpp2*^+/−^ mice are viable and harbor approximately half the systemic levels of LPA as compared to wild type mice (42, 69). Wild type and *Enpp2*^+/−^ mice were treated with anti-CD40, pI:C and OVA, an immunization protocol that has been shown to significantly increase the number of endogenous OVA-reactive CD8 T cells (52) and 4 days post treatment both genotypes were found to harbor equivalent frequencies of CD44+ H2-K^b^-OVA tetramer^+^ CD8 T cells (Figure 4A). To measure *in vivo* cytotoxic function of the activated endogenous OVA-specific CD8 T cells under homeostatic or reduced systemic LPA levels, i.v. transferred C57BL/6 (CD45.1) splenocytes were used as target cells after either no peptide treatment (unpulsed) or peptide treatment (pulsed) for 1 h at 37°C. The peptides used for target cell pulsing included an irrelevant HSV peptide or the high and low affinity SIINFEKL or SIIGFEKL OVA peptides, respectively. Additionally, transferred target cells were also differentially labeled with either low (unpulsed) or high (peptide pulsed) concentrations of eFluor670 prior to transfer into immunized hosts. One day later, spleens were harvested and the frequencies of irrelevant or antigen-specific peptide pulsed target cells were measured relative to unpulsed target cells (Figures 4B,C). The results from these experiments demonstrate that in *Enpp2*^+/−^ mice, where systemic LPA levels are approximately half the level of wild type (42, 69), *in vivo* CD8 T cell cytotoxicity is significantly increased approximately two-fold over cell killing in the presence of wild type LPA levels and to both relatively low and high affinity peptides (Figure 4D). These data provide confirmatory evidence that LPA acts *in vivo* to suppress antigen-specific CD8 T cell cytotoxicity and in response to endogenous lipid levels.

Further evidence that LPA_5_ signaling is indeed responsible for impaired *in vivo* CD8 T cell cytotoxicity was observed after implanting the syngeneic EL4 lymphoma variant, EG7 (53), into either C57BL/6, *Lpar5*^**−/−**^, or *Rag2*^−/−^ hosts and measuring lymphoma growth 12 days later. These results show that in *Rag2*^−/−^ hosts lacking an adaptive immune system, EG7 tumors grew significantly larger compare to tumor growth in the presence of a wild type immune system (Figure 4E). These results suggest that that anti-tumor adaptive immunity is able to provide some control of lymphoma growth. In contrast, EG7 lymphoma tumor growth was significantly reduced when implanted into LPA_5_-deficient hosts harboring an otherwise wild type endogenous adaptive immune system and relative to tumor growth observed in wild type hosts (Figure 4E). We also extended our previous findings to show that B16 melanoma growth remains significantly restrained by *Lpar5*^**−/−**^ tumor-specific CD8 T cells over 2 weeks after transfer [twice as long as previously shown (39)] and compared to the transfer of wild type tumor-specific CD8 T cells (Figure 4F). Together, these findings provide strong confirmatory evidence that LPA_5_ expressed by CD8 T cells signals normally *in vivo* to limit anti-tumor immunity.

### LPA_5_ Signaling Impairs TCR-Induced CD8 T Cell Granule Exocytosis

**Figure 5:**
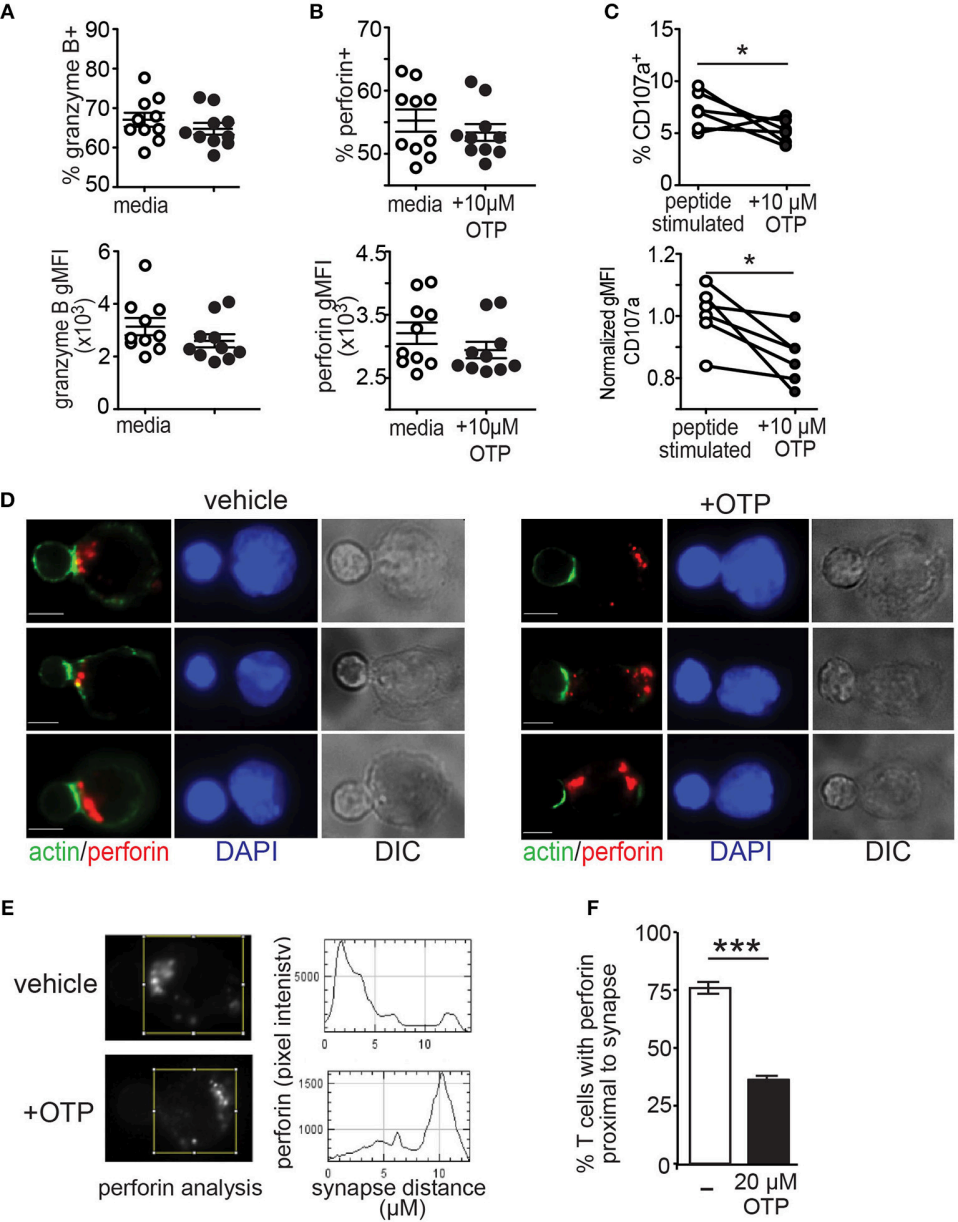
LPA suppresses granule exocytosis by effector CD8 T cells. **(A,B)** BFA-treated OT-I CD8 T cells were co-cultured with B16.cOVA.RFP for 4 h in the absence (open circles) or presence of 10 μM OTP (closed circles), fixed and stained for granzyme B **(A)** and perforin **(B)**. Top panels show frequency of granzyme B+ and perforin+ CD8 T cells and bottom panels show geometric mean fluorescence intensity (gMFI), respectively. Data are representative of two independent experiments with 10 technical controls per group. NS = *p* > 0.05 Student's *t-*test. **(C)** *in vitro* peptide-stimulated OT-I CD8 T cells stained for CD107a after 4 h in culture with B16.cOVA tumor cells at a 10:1 E:T ratio and either in the absence or presence of 10 μM OTP. Data is cumulative two-four experiments with technical triplicates. *p < 0.05 Student's *t-*test. **(D)** Effector OT-I CD8 T cell:SIINFEKL-pulsed B cell conjugates formed in the absence (left panels) or presence (right panels) of 20 μM OTP. Conjugates are stained with anti-perforin (red), phalloidin (green), and DAPI (blue). Representative conjugates are shown with perforin and actin (left) and DAPI alone (middle-left) staining, and differential interference contrast (DIC; middle-right). Scale bar = 5 μm. **(E)** Representative pixel intensity plots (y-axis) measuring perforin staining and distance from the synapse identified by polymerized actin (0 μm) and encompasses the entire cell for the two CD8 T cells shown in the top panels in **A**. **(F)** Summary of images acquired and analyzed in 3 independent experiments for a total of 46–50 cells per condition, ± SEM, *** *p* < 0.005 (Student's *t-*test). Each bar denotes the percent of conjugated OT-I T cells that display perforin localized to the proximal half of the T cell from the synapse.

Above, we show that the LPA_5_ expressed by CD8 T cells signals to suppress TCR-mediated intracellular calcium concentrations and ERK activation by both human and mouse CD8 T cells (Figures 1, 2). These two signaling effectors have previously been reported to be required for optimal granule exocytosis by CD8 T cells (56–61). Thus, we next addressed if LPA_5_ signaling impairs granule exocytosis and thereby contributed to the reduced CD8 T cell cytotoxicity. We initially measured the production of perforin and granzyme after 4 h of antigen-specific stimulation of OT-I CD8 T cells by co-culture with B16.cOVA.RFP cells alone or in the presence of 10 μM OTP (Figures 5A,B). These data revealed that while the expression of granzyme B and perforin, as well as frequency of granzyme B+ and perforin+ CD8 T cells, was slightly diminished in the presence of 10 μM OTP, this expression was not significantly changed relative to OT-I CD8 T cells stimulated in the absence of OTP. To directly assess granule exocytosis after antigen-specific activation of OT-I CD8 T cells, we measured the surface expression of CD107a, which serves as a surrogate marker for degranulation (70). These results showed that while TCR-activated OT-I CD8 T cells produced relatively normal amounts of perforin or granzyme in the presence of OTP (Figures 5A,B), 10 μM OTP significantly impaired the ability of CD8 T cells to export CD107a to the plasma membrane and, presumably, to release these effector molecules (Figure 5C). Thus, these results show that LPAR signaling on CD8 T cells following antigen-specific stimulation reduces CD107a surface expression suggesting that LPAR signaling impairs granule exocytosis.

Finally, to confirm LPAR signaling impairs granule exocytosis, activated antigen-specific OT-I CD8 T cells were added to SIINFEKL-pulsed purified splenic B cells and stable conjugates were stained for perforin, actin and DAPI to assess transport of perforin granules to the synapse using immunofluorescent microscopy. Perforin localization to the synapse was monitored 20 min after conjugate formation and in the absence or presence of the LPA_5_ selective LPA agonist, OTP (Figure 5D). Quantification of these results further demonstrated that perforin localization to the target cell interface was impaired in the presence of LPAR signaling (Figures 5E,F). Together, these results show that LPA_5_ signaling impairs granule exocytosis by effector CD8 T cells thus strongly indicating that LPA suppresses T cell cytotoxic activity.

## Discussion

LPA is a physiological lipid that signals via cognate GPCRs expressed by diverse cells types and we have previously documented that LPA engagement of LPA_5_ on B and T lymphocytes signals to negatively regulate antigen receptor signaling (39, 40). Importantly, the concentrations of LPA and the enzyme responsible for its extracellular production, ATX, have been reported to be increased in chronic inflammatory disorders such as chronic viral infections (26–28) autoimmune diseases (29, 30), obesity (31–33), and cancer (21, 23, 27, 34–38). In this report we have biochemically and functionally characterized how LPA signals via LPA_5_ to suppress CD8 T cell antigen receptor (TCR) signaling and cytotoxic activity. Specifically, we demonstrate that LPA_5_ is expressed by mouse and human CD8 T cells and in response to LPA signals to inhibit TCR-induced intracellular calcium mobilization, ERK activation and Nur77 expression. Importantly, this suppression of TCR signaling subsequently results in significantly impaired granule exocytosis due to impaired transport of cytotoxic granules to the target cell interface, a process dependent on intracellular calcium and ERK activation (56–61). As a result, in the presence of LPA both *in vitro* and *in vivo* antigen-specific cytotoxic activity is significantly compromised. Thus, LPA_5_ is an inhibitory G-protein coupled receptor expressed by CD8 T cells that suppresses T cell activation and cytolytic activity.

In this study we used CD8 T cells expressing a transgenic TCR to directly assess if and how LPA_5_ suppression of TCR signaling and function was dependent on antigen affinity. Our findings clearly demonstrate that when LPA (or its OTP analog) was present, TCR signaling and cytotoxic activity was blunted in response to both relatively high and low affinity antigens. However, these data also suggest that while TCR signaling in the presence of LPA was comparably repressed in response to both high and low affinity peptides (Figure 2), the killing activity of CD8 T cells was more severely inhibited in the presence of LPA when stimulated with the lower affinity SIIGFEKL peptide relative to the higher affinity SIINFEKL peptide (Figure 3). In this regard, it has been shown that TCR stimulation of CD8 effector cells leads to the recruitment of pERK to the immunological synapse and this recruitment is reduced and delayed with relatively weak peptide stimulation (71) ultimately leading to impaired delivery of lytic granules to the immunological synapse (72). Furthermore, the dependency on cytosolic calcium for granule exocytosis in primary NK cells has recently been accounted for by the requirement of calcium to associate with Munc13-4 (73), which subsequently promotes lytic granule fusion with the plasma membrane. Thus, these data together suggest inhibiting LPA signaling could lower the threshold of activation in response to both high and low affinity antigens permitting an increased repertoire of cytotoxic T cells to respond in the presence of high LPA levels induced by chronic inflammation.

We find LPA_5_ is constitutively expressed by naïve and effector CD8 T cells and further show that LPA_5_ signaling suppresses both TCR signaling and effector CD8 T cell killing activity, respectively. In contrast, CTLA-4, PD-1 and other inhibitory receptors (e.g., LAG-3, TIM-3, and TIGIT) are not normally expressed by naïve CD8 T cells but instead their expression is induced upon TCR-mediated cell activation (17, 18). Thus, current checkpoint blockade therapy appears to provide benefit by relieving inhibition of effector CD8 T cells and promoting cytotoxic activity. However, the ability of a pathogen or tumor-specific naïve CD8 T cell to become activated, differentiate and exert effector function has been shown to be critically dependent on the ability of the TCR to not only recognize cognate antigen but to also transmit signals as a result of this cognate recognition. Thus, the initial TCR recognition and subsequent signaling is crucial for appropriate CD8 T cell activation, robust proliferation and differentiation into memory and effector cytotoxic T lymphocytes (CTLs) able to eliminate infected cells or nascent tumors (74–77). In consideration of our findings, this suggests that the therapeutic *in vivo* inhibition of LPA-LPA_5_ signaling would not only enhance CD8 T cell effector function but also provide the added benefit of lowering the threshold of TCR-mediated activation of naïve CD8 T cells recognizing low-affinity antigens typical of (non-mutated) tumor antigens.

LPA has been reported in serum at high nanomolar to low micromolar levels in healthy individuals and wild type mice (22, 78) but can be significantly increased in inflammatory settings. However, beyond changes in LPA concentration at the systemic level, local production of LPA in certain environments critically regulates immune cell function under homeostatic as well as inflammatory conditions. *Enpp2*, the gene encoding ATX, is constitutively expressed under homeostatic conditions by both endothelial cells of high endothelial venules (HEVs) (79, 80) and fibroblastic reticular cells (FRCs) within secondary lymphoid organs (81). The ATX produced by these cell types associates with integrins on the surface of these cells and produces LPA that subsequently signals locally via LPA_2_ on T cells to promote transendothelial migration and intranodal motility, respectively (82–85). Importantly, a recent report has revealed that HEV Enpp2 expression decreases upon antigenic stimulation (86) suggesting that HEV-derived ATX, and the subsequently produced LPA, is modulated during infection to regulate T cell entry into secondary lymphoid organs and to enhance activation of T cells recognizing both high and low affinity antigens. Indeed, we find here that when levels of LPA are halved, as in *Enpp2*^−/+^ mice (42, 69), *in vivo* CD8 T cell cytotoxic activity is increased by 2-fold in response to both relatively high and low affinity antigens (Figure 4D). These findings indicate that LPA_5_ signaling specifically restrains cytotoxic function by wild type CD8 T cells. This conclusion is further supported by the ability of *Lpar5*^−/−^ mice and *Lpar5*^−/−^ tumor-specific CD8 T cells to better control EG7 lymphoma and B16 melanoma growth, respectively, compared to wild type (Figures 4E,F). Thus, the regulation of LPA concentrations by specific cell types are modulated upon inflammation to typically promote an effective immune response. Nevertheless, in response to certain chronic inflammatory conditions (e.g., specific cancers and viral infections), our findings indicate that specifically targeting LPA_5_ signaling within secondary lymphoid organs could enhance activation of a broader repertoire of CD8 T cells to promote a more effective immune response.

CTLA-4 is considered to attenuate T cell responses by precluding CD28 stimulatory co-receptor signaling through the competitive binding of CD80/CD86 (B7-1/B7-2) receptor ligands. In contrast, the PD-1 inhibitory receptor, whose ligands are PD-L1 and PD-L2, harbors an immune receptor inhibitory tyrosine motif (ITIM) that inhibits TCR and CD28 signaling by the recruitment and activity of the SHP-2 tyrosine phosphatase (87–89) although recent findings indicate that PD-1 may initially suppress CD28 co-stimulatory signaling (88). In B cells, we have characterized LPA_5_ inhibitory signaling to proceed via a Gα_12/13_-ARHGEF1 signaling axis to impede BCR-induced IP_3_ receptor activity and subsequent intracellular calcium stores release. Whether a similar LPA_5_ signaling pathway operates in human and mouse CD8 T cells is currently under investigation. In mouse and human CD8 T cells, we demonstrate LPA_5_ signaling also suppresses intracellular calcium mobilization and ERK activation and as a result granule exocytosis and cytotoxic activity are depressed. This LPA_5_-mediated inhibitory pathway clearly differs from mechanisms used by either CTLA-4 or PD-1 and, given the clinical success of small molecule antagonism of GPCRs, indicates that interfering with *in vivo* LPA_5_ signaling may provide additional therapeutic benefit against chronic infections and diverse malignancies. Our findings would further predict that small molecule antagonism of the ATX-LPA-LPA_5_ axis may be useful to reinvigorate exhausted T cells during chronic infections or cancer.

## Data Availability

All datasets generated for this study are included in the manuscript and/or the Supplementary Files.

## Ethics Statement

All procedures with animals were approved by the University of Colorado Institutional Animal Care and Use Committee.

All human studies were performed in compliance with University of Colorado Institutional Review Boards and in accordance with the Declaration of Helsinki.

## Author Contributions

RT, DM, KK, and RP designed the research studies. DM and KK conducted the experiments and acquired data with assistance from PS. RT, DM, RP, and KK analyzed data and/or interpreted results. GT provided reagents. RT, DM, KK, and RP wrote the manuscript.

### Conflict of Interest Statement

GT is founder, consultant and stock holder in RxBio Inc., that has licensed intellectual properties pertinent to OTP from the University of Tennessee Research Foundation. The remaining authors declare that the research was conducted in the absence of any commercial or financial relationships that could be construed as potential conflict of interest.

## Supplementary Material

Supplemental Figure 1 LPA_5_ selectively signals to negatively regulate CD8 T cell TCR signaling. **(A)** Intracellular calcium mobilization in C57BL/6, *Lpar5*^**−/−**^, *Lpar6*^**−/−**^, or *Lpar6*^**−/−**^ CD8 T cells after anti-CD3 (arrow) stimulation alone (red) or in the presence of 10 μM LPA (blue). Calcium was measured in indo1-loaded splenocytes stained with anti-CD8 and biotin conjugated anti-CD3, followed by CD3 cross-linking with 10 μg/mL avidin. Data are representative of 3 independent experiments each. **(B)** Cell viability of day 6 activated OT-I CD8 T cells plated in media alone or with the indicated amounts of OTP normalized to media only samples. Data is representative of two independent experiments with 3 technical replicates per group. **(C)** CD69 surface expression by OT-I T cells after 24 h in culture with varying concentrations of SIINFEKL peptide and in the absence (black) or presence of 10uM (red) or 20 μM (blue) OTP. ^***^*p* < 0.0005 as determined by two-way ANOVA. Data are representative of 2 independent experiments with 6 technical replicates per group. ^***^*p* < 0.0005 as determined by two-way ANOVA.

Supplemental Figure 2 *in vitro* CD8 T cell cytotoxicity assay. **(A)** RFP expression by B16.F10 parental cells (red) and lentiviral transduced B16.F10 cells expressing RFP under the EF-1α promoter (blue). **(B)** Microscope image and identification of transduced viable (red), apoptotic B16.F10 cells [purple = red + green (caspase 3/7+)] and apoptotic CD8 T cells (green). **(C)** Normalized tumor cell death after addition of peptide activated OT-I T cells at varying effector to target cell ratios using 1 × 10^4^ B16.cOVA.RFP tumor cells in all conditions. ^*^*p* < 0.005 or ^***^*p* < 0.0005 using a two-way ANOVA with, 4 images collected per will with technical triplicates per condition. Area under the curve was calculated for each respective E:T ratio. ^*^*p* < 0.05 or ^***^*p* < 0.0005 using Student *t-*test. **(D)** Naïve or anti-CD3 activated C57BL/6, P14, or OT-I CD8 T cells were added to B16.cOVA tumor cells at a E:T ratio of 10:1. P14 CD8 T cells harbor a transgenic TCR specific for an irrelevant viral antigen and thus are not tumor-specific and do not kill B16 targets. ^***^*p* < 0.0005 using a two-way ANOVA with, 4 images collected per well with technical triplicates per condition. **(E)** Area under the curve was calculated for each respective condition in panel **D**. ^***^*p* < 0.0005 using Student *t-*test. Data are representative of 2 independent experiments. **(F)** B16.F10.RFP melanoma cells were plated in media (0 μM) or with increasing concentration of OTP together with an indicator of active Caspase 3/7. As a positive control, day 6 activated effector T cells (filled black circles) were plated to measure active apoptosis. Four images of each technical triplicate were measured every 2 h for 24 h. Data is accumulation of two independent experiments.
