# Supplementary figures and images for: LPA5 Is an Inhibitory Receptor That Suppresses CD8 T-Cell Cytotoxic Function via Disruption of Early TCR Signaling
Source: Front Immunol. 2019 May 28;10:1159. doi: 10.3389/fimmu.2019.01159 (PMC6558414; doi:10.3389/fimmu.2019.01159)

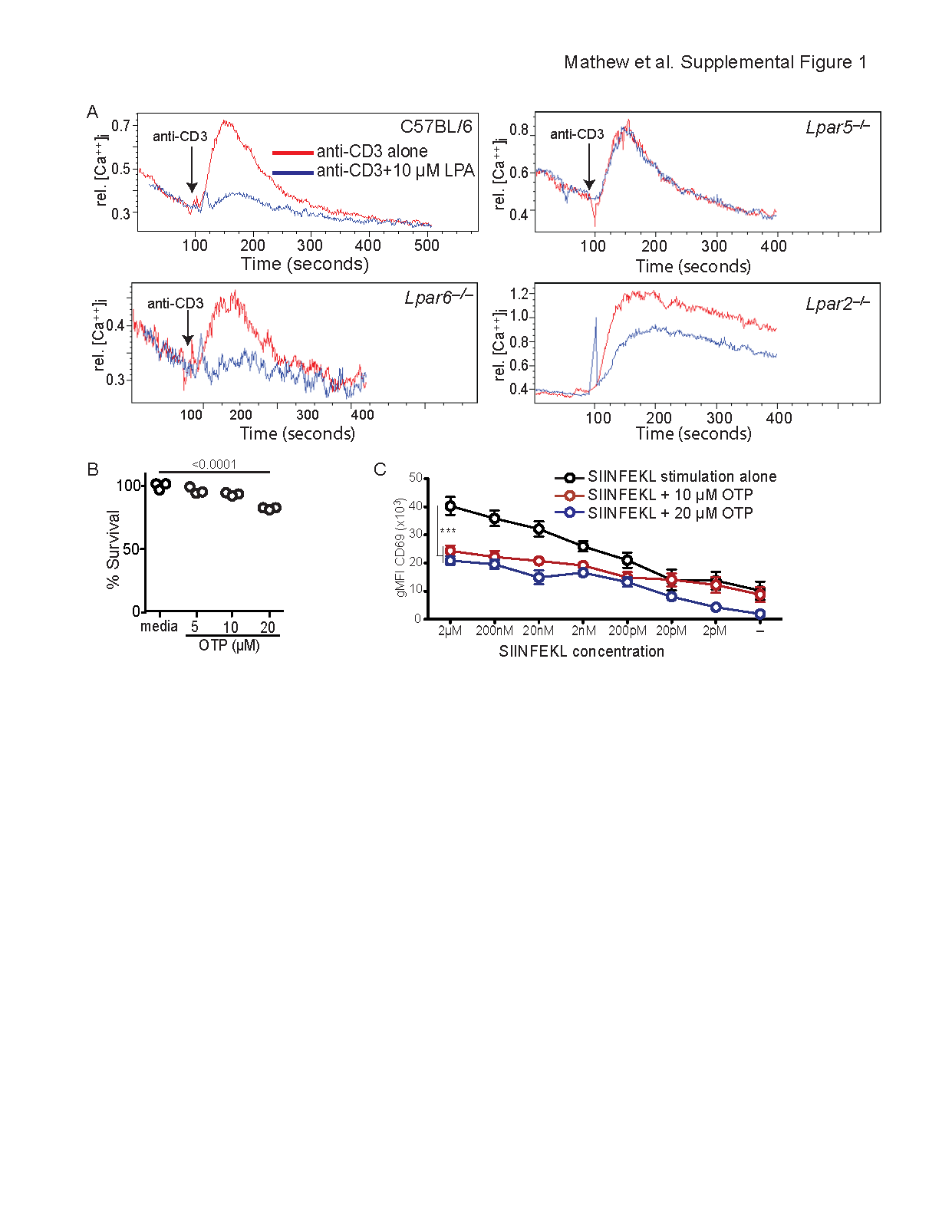

Supplement: Supplemental Figure 1 — LPA5 selectively signals to negatively regulate CD8 T cell TCR signaling. (A) Intracellular calcium mobilization in C57BL/6, Lpar5−/−, Lpar6−/−, or Lpar6−/− CD8 T cells after anti-CD3 (arrow) stimulation alone (red) or in the presence of 10 μM LPA (blue). Calcium was measured in indo1-loaded splenocytes stained with anti-CD8 and biotin conjugated anti-CD3, followed by CD3 cross-linking with 10 μg/mL avidin. Data are representative of 3 independent experiments each. (B) Cell viability of day 6 activated OT-I CD8 T cells plated in media alone or with the indicated amounts of OTP normalized to media only samples. Data is representative of two independent experiments with 3 technical replicates per group. (C) CD69 surface expression by OT-I T cells after 24 h in culture with varying concentrations of SIINFEKL peptide and in the absence (black) or presence of 10uM (red) or 20 μM (blue) OTP. ***p < 0.0005 as determined by two-way ANOVA. Data are representative of 2 independent experiments with 6 technical replicates per group. ***p < 0.0005 as determined by two-way ANOVA. [file Image_1.tiff]

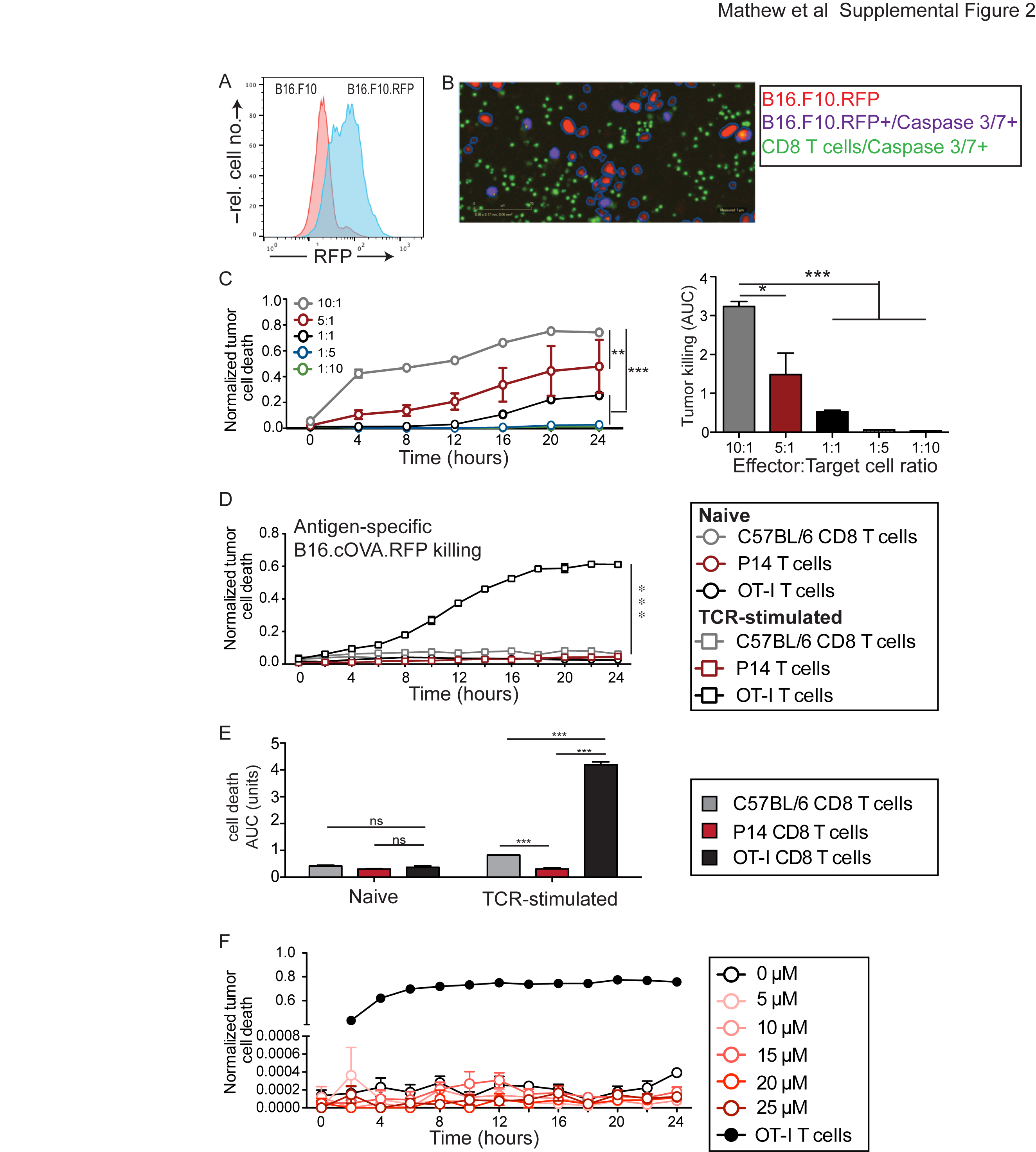

Supplement: Supplemental Figure 2 — in vitro CD8 T cell cytotoxicity assay. (A) RFP expression by B16.F10 parental cells (red) and lentiviral transduced B16.F10 cells expressing RFP under the EF-1α promoter (blue). (B) Microscope image and identification of transduced viable (red), apoptotic B16.F10 cells [purple = red + green (caspase 3/7+)] and apoptotic CD8 T cells (green). (C) Normalized tumor cell death after addition of peptide activated OT-I T cells at varying effector to target cell ratios using 1 × 10^4^ B16.cOVA.RFP tumor cells in all conditions. *p < 0.005 or ***p < 0.0005 using a two-way ANOVA with, 4 images collected per will with technical triplicates per condition. Area under the curve was calculated for each respective E:T ratio. *p < 0.05 or ***p < 0.0005 using Student t-test. (D) Naïve or anti-CD3 activated C57BL/6, P14, or OT-I CD8 T cells were added to B16.cOVA tumor cells at a E:T ratio of 10:1. P14 CD8 T cells harbor a transgenic TCR specific for an irrelevant viral antigen and thus are not tumor-specific and do not kill B16 targets. ***p < 0.0005 using a two-way ANOVA with, 4 images collected per well with technical triplicates per condition. (E) Area under the curve was calculated for each respective condition in panel D. ***p < 0.0005 using Student t-test. Data are representative of 2 independent experiments. (F) B16.F10.RFP melanoma cells were plated in media (0 μM) or with increasing concentration of OTP together with an indicator of active Caspase 3/7. As a positive control, day 6 activated effector T cells (filled black circles) were plated to measure active apoptosis. Four images of each technical triplicate were measured every 2 h for 24 h. Data is accumulation of two independent experiments. [file Image_2.TIF]
